# (*E*)-1-[2-Hy­droxy-4,6-bis­(meth­oxy­meth­oxy)phen­yl]-3-[3-meth­oxy-4-(meth­oxy­meth­oxy)phen­yl]prop-2-en-1-one

**DOI:** 10.1107/S1600536811041213

**Published:** 2011-10-12

**Authors:** Liu-Shuan Chang, Chen-Yang Li, Yan-Mei Zhao, Fang Xu, Zheng-Yi Gu

**Affiliations:** a Department of Military Protective Medicine, Logistics College of Chinese People’s Armed Police Forces, Tianjin 300162, People’s Republic of China; b Xinjiang Institute of Materia Medica, Xinjiang 830004, People’s Republic of China

## Abstract

The title compound, C_22_H_26_O_9_, crystallizes with two independent mol­ecules in the asymmetric unit in which the dihedral angles between the two benzene rings are 21.4 (2) and 5.1 (2)°. An intra­molecular O—H⋯O hydrogen bond occurs in each mol­ecule. Inter­molecular C—H⋯O hydrogen bonds stabilize the crystal structure.

## Related literature

For the biological activity of flavonoids, see: Jung *et al.* (2006 ▶); Ong & Khoo (1996 ▶); Vessal *et al.* (2003 ▶); Sousa *et al.* (2004 ▶). For bond-length data, see: Allen *et al.* (1987 ▶); Chu *et al.* (2004 ▶); Zhang *et al.* (2011 ▶). For the preparation, see: Duan *et al.* (2006 ▶).
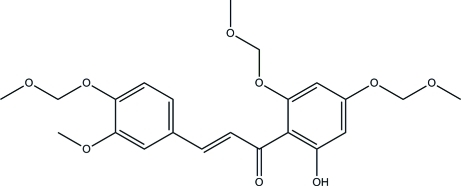


## Experimental

### 

#### Crystal data

C_22_H_26_O_9_
*M* *_r_* = 434.43
Monoclinic, 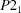

*a* = 12.008 (3) Å
*b* = 13.016 (4) Å
*c* = 13.663 (4) Å
β = 97.154 (4)°
*V* = 2119.0 (10) Å^3^
*Z* = 4
Mo *K*α radiation
μ = 0.11 mm^−1^
*T* = 113 K
0.24 × 0.22 × 0.18 mm

#### Data collection

Rigaku Saturn CCD area-detector diffractometer
Absorption correction: multi-scan (*CrystalClear*; Rigaku/MSC, 2009) ▶ *T*_min_ = 0.975, *T*_max_ = 0.981
22288 measured reflections
5259 independent reflections
4715 reflections with *I* > 2σ(*I*)
*R*_int_ = 0.045

#### Refinement

*R*[*F* ^2^ > 2σ(*F* ^2^)] = 0.038
*wR*(*F* ^2^) = 0.076
*S* = 1.03
5259 reflections
569 parameters
1 restraint
H-atom parameters constrained
Δρ_max_ = 0.21 e Å^−3^
Δρ_min_ = −0.18 e Å^−3^

### 

Data collection: *CrystalClear-SM Expert* (Rigaku/MSC, 2009) ▶; cell refinement: *CrystalClear-SM Expert* ▶; data reduction: *CrystalClear-SM Expert* ▶; program(s) used to solve structure: *SHELXS97* (Sheldrick, 2008 ▶); program(s) used to refine structure: *SHELXL97* (Sheldrick, 2008 ▶); molecular graphics: *SHELXTL* (Sheldrick, 2008 ▶); software used to prepare material for publication: *SHELXTL*.

## Supplementary Material

Crystal structure: contains datablock(s) I, global. DOI: 10.1107/S1600536811041213/hg5103sup1.cif

Structure factors: contains datablock(s) I. DOI: 10.1107/S1600536811041213/hg5103Isup2.hkl

Supplementary material file. DOI: 10.1107/S1600536811041213/hg5103Isup3.cml

Additional supplementary materials:  crystallographic information; 3D view; checkCIF report

## Figures and Tables

**Table 1:** Hydrogen-bond geometry (Å, °)

*D*—H⋯*A*	*D*—H	H⋯*A*	*D*⋯*A*	*D*—H⋯*A*
O5—H5⋯O6	0.84	1.75	2.506 (2)	148
O14—H14⋯O15	0.84	1.72	2.475 (2)	148
C8—H8*C*⋯O8^i^	0.98	2.57	3.312 (3)	132
C9—H9*A*⋯O5^ii^	0.99	2.52	3.444 (3)	155

Symmetry codes: (i) 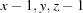; (ii) 
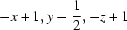
.

**Fig. 1.**
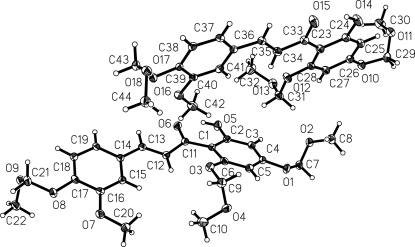
View of the title compound, with displacement ellipsoids drawn at the 40% probability level.

## supplementary crystallographic information

### Comment

Several flavonoids, such as Hesperidin and naringin(Jung *et al.*, 2006), myricetin (Ong & Khoo, 1996), quercetin (Vessal *et al.*, 2003), Kaempferol-3,7-O-(*r*)-dirhamnoside (Sousa *et al.*, 2004), have been reported to treat diabetes. We synthesized a series of 5,7-dihydroxy flavonoids. The vitro antidiabetic activity experiment showed that most of the flavonoids showed a remarkable *in vitro* anti-diabetic activity. The title compound, (*E*)-1-(2-hydroxy-4,6-bis(methoxymethoxy)phenyl)-3- (3-methoxy-4-(methoxymethoxy)phenyl)prop-2-en-1-one was prepared as an intermediate.

In title compound, C_22_H_26_O_9_, crystallizes with two independent molecules in the asymmetric unit. All bond lengths and angles in the molecular are normal (Allen *et al.*, 1987) and in a good agreement with those reported previously (Chu *et al.*, 2004; Zhang *et al.*, 2011). The dihedral angle between two phenyl ring (C1—C6 and C14—C19; C23—C28 and C36—C41) are 21.4 (2)° and 5.1 (2) °, respectively. The C—H···O intermolecular hydrogen bonds stabilized the crystal structure (Table 1).

### Experimental

A round-bottomed flask was charged with 1.52 g (10 mmol) of 4-hydroxy-3-methoxybenzaldehyde, 9.66 g (70 mmol) of K_2_CO_3_, 805 mg (10 mmol) of chloromethyl methyl ether (MOMCl) and 30 ml of anhydrous acetone, and the mixture was stirred at room temperature for four hours. The reaction mixture was filtered, and evaporated to afford 3-methoxy-4-(methoxymethoxy)benzaldehyde. Dropwise chloromethyl methyl ether (4.83 g, 6 mmol) was added to a mixture of 2,4,6-trihydroxyacetophenone (503.0 mg, 3 mmol) and K_2_CO_3_ (2.89 g, 21 mmol) in anhydrous acetone (30 ml). The mixture was heated at reflux for 1.5 h, filtered, and evaporated to afford 2-hydroxy-4,6-dimethoxymethoxyacetophenone. Then 3-methoxy-4-(methoxymethoxy)benzaldehyde (589 mg, 3.0 mmol) in 60% NaOH aqua (8 ml) and MeOH (15 ml) was added and stirred for 24 h. The resulting mixture was poured into cold 2 *M* HCl (40 ml), and then extracted with three 20-ml portions of EtOAc. The organic layer was washed with water and saturated brine, dried over MgSO4, and evaporated to afford the title compound *via* recrystallization from EtOH (Duan *et al.*, 2006). Single crystals suitable for X-ray diffraction were obtained from slow evaporation of a solution of the pure title compound in ethanol at room temperature.

### Refinement

All H atoms were found on difference maps, with C—H = 0.95–0.99, O—H = 0.84 Å and included in the final cycles of refinement using a riding model, with *U*_iso_(H) = 1.2*U*_eq_(C) and 1.5*U*_eq_(C, O) for the methyl and hydroxyl H atoms.

### Crystal data

**Table:**

C_22_H_26_O_9_	*F*(000) = 920
*M**_r_* = 434.43	*D*_x_ = 1.362 Mg m^−^^3^
Monoclinic, *P*2_1_	Mo *K*α radiation, λ = 0.71073 Å
Hall symbol: P 2yb	Cell parameters from 7434 reflections
*a* = 12.008 (3) Å	θ = 1.7–27.9°
*b* = 13.016 (4) Å	µ = 0.11 mm^−^^1^
*c* = 13.663 (4) Å	*T* = 113 K
β = 97.154 (4)°	Block, orange
*V* = 2119.0 (10) Å^3^	0.24 × 0.22 × 0.18 mm
*Z* = 4	

### Data collection

**Table:**

Rigaku Saturn CCD area-detector diffractometer	5259 independent reflections
Radiation source: rotating anode	4715 reflections with *I* > 2σ(*I*)
multilayer	*R*_int_ = 0.045
Detector resolution: 14.63 pixels mm^-1^	θ_max_ = 27.9°, θ_min_ = 1.7°
ω and φ scans	*h* = −14→15
Absorption correction: multi-scan (*CrystalClear*; Rigaku/MSC, 2009)	*k* = −17→17
*T*_min_ = 0.975, *T*_max_ = 0.981	*l* = −17→17
22288 measured reflections	

### Refinement

**Table:**

Refinement on *F*^2^	Primary atom site location: structure-invariant direct methods
Least-squares matrix: full	Secondary atom site location: difference Fourier map
*R*[*F*^2^ > 2σ(*F*^2^)] = 0.038	Hydrogen site location: inferred from neighbouring sites
*wR*(*F*^2^) = 0.076	H-atom parameters constrained
*S* = 1.03	*w* = 1/[σ^2^(*F*_o_^2^) + (0.0347*P*)^2^] where *P* = (*F*_o_^2^ + 2*F*_c_^2^)/3
5259 reflections	(Δ/σ)_max_ = 0.001
569 parameters	Δρ_max_ = 0.21 e Å^−^^3^
1 restraint	Δρ_min_ = −0.18 e Å^−^^3^

### Special details

**Table:**

Geometry. All e.s.d.'s (except the e.s.d. in the dihedral angle between two l.s. planes) are estimated using the full covariance matrix. The cell e.s.d.'s are taken into account individually in the estimation of e.s.d.'s in distances, angles and torsion angles; correlations between e.s.d.'s in cell parameters are only used when they are defined by crystal symmetry. An approximate (isotropic) treatment of cell e.s.d.'s is used for estimating e.s.d.'s involving l.s. planes.
Refinement. Refinement of *F*^2^ against ALL reflections. The weighted *R*-factor *wR* and goodness of fit *S* are based on *F*^2^, conventional *R*-factors *R* are based on *F*, with *F* set to zero for negative *F*^2^. The threshold expression of *F*^2^ > σ(*F*^2^) is used only for calculating *R*-factors(gt) *etc*. and is not relevant to the choice of reflections for refinement. *R*-factors based on *F*^2^ are statistically about twice as large as those based on *F*, and *R*- factors based on ALL data will be even larger.

### Fractional atomic coordinates and isotropic or equivalent isotropic displacement parameters (Å^2^)

**Table:**

	*x*	*y*	*z*	*U*_iso_*/*U*_eq_	
O1	0.22434 (13)	0.90191 (12)	0.50952 (12)	0.0273 (4)	
O2	0.14482 (13)	0.95752 (11)	0.35274 (11)	0.0244 (4)	
O3	0.58068 (13)	0.88250 (11)	0.69967 (11)	0.0249 (4)	
O4	0.46961 (13)	0.78830 (13)	0.79761 (12)	0.0312 (4)	
O5	0.48367 (14)	1.17704 (12)	0.49837 (12)	0.0302 (4)	
H5	0.5482	1.1951	0.5233	0.045*	
O6	0.66802 (13)	1.16766 (12)	0.60636 (11)	0.0267 (4)	
O7	0.91164 (14)	0.91335 (12)	1.08562 (11)	0.0273 (4)	
O8	1.09693 (14)	1.01811 (12)	1.11830 (11)	0.0257 (4)	
O9	1.19143 (13)	1.17603 (12)	1.14491 (11)	0.0266 (4)	
O10	0.18533 (13)	0.81450 (12)	0.00995 (11)	0.0265 (4)	
O11	0.15303 (14)	0.77679 (11)	−0.15985 (12)	0.0275 (4)	
O12	0.49745 (13)	0.80118 (12)	0.25640 (11)	0.0273 (4)	
O13	0.44653 (14)	0.97448 (11)	0.26498 (12)	0.0296 (4)	
O14	0.46845 (14)	0.56582 (13)	−0.01679 (12)	0.0329 (4)	
H14	0.5328	0.5489	0.0095	0.049*	
O15	0.64358 (14)	0.57331 (13)	0.09948 (12)	0.0360 (4)	
O16	0.89706 (13)	0.82460 (12)	0.57367 (11)	0.0248 (4)	
O17	1.08189 (13)	0.71974 (11)	0.60496 (11)	0.0233 (4)	
O18	1.18426 (13)	0.56566 (12)	0.63086 (11)	0.0266 (4)	
C1	0.53421 (18)	1.03548 (16)	0.60884 (15)	0.0195 (5)	
C2	0.45869 (19)	1.08459 (16)	0.53524 (16)	0.0213 (5)	
C3	0.35580 (19)	1.04253 (17)	0.49855 (16)	0.0222 (5)	
H3	0.3074	1.0772	0.4489	0.027*	
C4	0.32511 (19)	0.94969 (17)	0.53531 (16)	0.0225 (5)	
C5	0.39895 (19)	0.89474 (17)	0.60430 (16)	0.0244 (5)	
H5A	0.3777	0.8297	0.6274	0.029*	
C6	0.50153 (18)	0.93550 (17)	0.63790 (16)	0.0218 (5)	
C7	0.13674 (19)	0.95890 (18)	0.45297 (17)	0.0255 (5)	
H7A	0.0633	0.9298	0.4645	0.031*	
H7B	0.1394	1.0310	0.4762	0.031*	
C8	0.1145 (2)	0.85967 (17)	0.30924 (18)	0.0297 (6)	
H8A	0.0405	0.8394	0.3259	0.045*	
H8B	0.1703	0.8082	0.3347	0.045*	
H8C	0.1120	0.8645	0.2374	0.045*	
C9	0.5511 (2)	0.78293 (17)	0.73444 (17)	0.0277 (5)	
H9A	0.5233	0.7394	0.6772	0.033*	
H9B	0.6190	0.7498	0.7692	0.033*	
C10	0.5096 (2)	0.8409 (2)	0.8875 (2)	0.0481 (8)	
H10A	0.5307	0.9113	0.8724	0.072*	
H10B	0.5751	0.8047	0.9210	0.072*	
H10C	0.4501	0.8426	0.9306	0.072*	
C11	0.63687 (19)	1.08955 (17)	0.64962 (16)	0.0214 (5)	
C12	0.70300 (18)	1.05763 (16)	0.74328 (16)	0.0211 (5)	
H12	0.6728	1.0086	0.7842	0.025*	
C13	0.80475 (18)	1.09697 (16)	0.77090 (16)	0.0207 (5)	
H13	0.8301	1.1467	0.7278	0.025*	
C14	0.88206 (18)	1.07296 (17)	0.85929 (16)	0.0203 (5)	
C15	0.85724 (19)	0.99989 (16)	0.92922 (16)	0.0212 (5)	
H15	0.7888	0.9626	0.9184	0.025*	
C16	0.9308 (2)	0.98154 (16)	1.01337 (16)	0.0205 (5)	
C17	1.03260 (19)	1.03632 (16)	1.03000 (16)	0.0211 (5)	
C18	1.05886 (19)	1.10596 (16)	0.95928 (16)	0.0221 (5)	
H18	1.1287	1.1411	0.9683	0.027*	
C19	0.98402 (18)	1.12444 (17)	0.87596 (16)	0.0222 (5)	
H19	1.0026	1.1734	0.8290	0.027*	
C20	0.8059 (2)	0.86135 (18)	1.07342 (18)	0.0313 (6)	
H20A	0.7979	0.8226	1.0114	0.047*	
H20B	0.7451	0.9118	1.0718	0.047*	
H20C	0.8021	0.8140	1.1287	0.047*	
C21	1.2022 (2)	1.07013 (18)	1.13626 (17)	0.0269 (5)	
H21A	1.2443	1.0430	1.1977	0.032*	
H21B	1.2466	1.0551	1.0815	0.032*	
C22	1.1447 (2)	1.20680 (19)	1.23220 (17)	0.0318 (6)	
H22A	1.1861	1.1735	1.2899	0.048*	
H22B	1.0656	1.1863	1.2265	0.048*	
H22C	1.1505	1.2816	1.2397	0.048*	
C23	0.49240 (19)	0.68245 (17)	0.12260 (16)	0.0224 (5)	
C24	0.42976 (19)	0.64374 (17)	0.03440 (16)	0.0230 (5)	
C25	0.32671 (19)	0.68403 (17)	−0.00400 (16)	0.0236 (5)	
H25	0.2863	0.6555	−0.0619	0.028*	
C26	0.28369 (19)	0.76586 (17)	0.04285 (16)	0.0227 (5)	
C27	0.34081 (18)	0.80713 (17)	0.12906 (16)	0.0228 (5)	
H27	0.3101	0.8640	0.1600	0.027*	
C28	0.44134 (18)	0.76566 (16)	0.16925 (16)	0.0215 (5)	
C29	0.11434 (19)	0.76794 (18)	−0.06884 (17)	0.0265 (5)	
H29A	0.1061	0.6941	−0.0537	0.032*	
H29B	0.0390	0.7997	−0.0729	0.032*	
C30	0.1521 (2)	0.88016 (18)	−0.19537 (19)	0.0395 (7)	
H30A	0.2100	0.9201	−0.1551	0.059*	
H30B	0.0784	0.9109	−0.1912	0.059*	
H30C	0.1674	0.8803	−0.2642	0.059*	
C31	0.4462 (2)	0.87896 (17)	0.31042 (17)	0.0269 (5)	
H31A	0.3678	0.8589	0.3164	0.032*	
H31B	0.4871	0.8839	0.3778	0.032*	
C32	0.5560 (2)	1.0166 (2)	0.2683 (2)	0.0408 (7)	
H32A	0.6041	0.9689	0.2373	0.061*	
H32B	0.5875	1.0279	0.3372	0.061*	
H32C	0.5521	1.0822	0.2328	0.061*	
C33	0.60378 (19)	0.63850 (17)	0.15390 (16)	0.0235 (5)	
C34	0.67431 (19)	0.66787 (17)	0.24565 (17)	0.0243 (5)	
H34	0.6443	0.7116	0.2915	0.029*	
C35	0.77910 (18)	0.63422 (16)	0.26537 (16)	0.0207 (5)	
H35	0.8046	0.5895	0.2180	0.025*	
C36	0.85976 (18)	0.65812 (16)	0.35166 (16)	0.0191 (5)	
C37	0.96138 (18)	0.60702 (16)	0.36697 (16)	0.0210 (5)	
H37	0.9787	0.5577	0.3199	0.025*	
C38	1.03903 (18)	0.62596 (17)	0.44958 (16)	0.0206 (5)	
H38	1.1084	0.5900	0.4582	0.025*	
C39	1.01467 (18)	0.69756 (16)	0.51928 (16)	0.0192 (5)	
C40	0.91248 (18)	0.75263 (15)	0.50337 (16)	0.0190 (5)	
C41	0.83640 (19)	0.73288 (16)	0.42099 (16)	0.0204 (5)	
H41	0.7678	0.7700	0.4110	0.024*	
C42	0.7928 (2)	0.87817 (18)	0.56172 (17)	0.0300 (6)	
H42A	0.7310	0.8287	0.5595	0.045*	
H42B	0.7859	0.9175	0.5001	0.045*	
H42C	0.7898	0.9251	0.6174	0.045*	
C43	1.18969 (18)	0.67200 (18)	0.61951 (17)	0.0249 (5)	
H43A	1.2300	0.6875	0.5623	0.030*	
H43B	1.2336	0.7018	0.6789	0.030*	
C44	1.1416 (2)	0.53416 (19)	0.71952 (18)	0.0299 (6)	
H44A	1.0617	0.5514	0.7151	0.045*	
H44B	1.1827	0.5698	0.7761	0.045*	
H44C	1.1513	0.4598	0.7280	0.045*	

### Atomic displacement parameters (Å^2^)

**Table:**

	*U*^11^	*U*^22^	*U*^33^	*U*^12^	*U*^13^	*U*^23^
O1	0.0205 (9)	0.0293 (9)	0.0301 (9)	−0.0058 (7)	−0.0044 (7)	0.0054 (7)
O2	0.0271 (9)	0.0210 (8)	0.0239 (9)	−0.0014 (7)	−0.0013 (7)	0.0003 (6)
O3	0.0236 (9)	0.0231 (8)	0.0272 (9)	0.0009 (6)	−0.0002 (7)	0.0071 (6)
O4	0.0299 (9)	0.0339 (9)	0.0309 (10)	−0.0061 (8)	0.0076 (8)	0.0061 (7)
O5	0.0312 (10)	0.0249 (8)	0.0324 (10)	−0.0075 (7)	−0.0049 (8)	0.0089 (7)
O6	0.0249 (9)	0.0274 (8)	0.0272 (9)	−0.0059 (7)	0.0009 (7)	0.0063 (7)
O7	0.0338 (10)	0.0255 (9)	0.0217 (9)	−0.0054 (7)	0.0003 (8)	0.0053 (7)
O8	0.0266 (9)	0.0271 (9)	0.0215 (9)	−0.0012 (7)	−0.0046 (7)	0.0001 (7)
O9	0.0287 (9)	0.0263 (8)	0.0242 (9)	−0.0023 (7)	0.0012 (7)	−0.0024 (7)
O10	0.0210 (9)	0.0304 (9)	0.0266 (9)	0.0054 (7)	−0.0035 (7)	−0.0024 (7)
O11	0.0361 (10)	0.0220 (8)	0.0234 (9)	0.0031 (7)	−0.0002 (7)	0.0016 (7)
O12	0.0292 (9)	0.0286 (9)	0.0227 (8)	0.0087 (7)	−0.0023 (7)	−0.0068 (7)
O13	0.0269 (10)	0.0278 (9)	0.0332 (10)	0.0035 (7)	−0.0001 (8)	−0.0038 (7)
O14	0.0334 (11)	0.0320 (9)	0.0309 (10)	0.0127 (8)	−0.0054 (8)	−0.0098 (8)
O15	0.0318 (10)	0.0381 (10)	0.0354 (10)	0.0125 (8)	−0.0064 (8)	−0.0142 (8)
O16	0.0291 (9)	0.0213 (8)	0.0248 (9)	0.0044 (7)	0.0060 (7)	−0.0048 (7)
O17	0.0226 (9)	0.0259 (8)	0.0202 (8)	0.0005 (7)	−0.0015 (7)	−0.0016 (6)
O18	0.0278 (10)	0.0280 (9)	0.0239 (9)	0.0033 (7)	0.0023 (7)	0.0001 (7)
C1	0.0197 (12)	0.0213 (11)	0.0176 (11)	−0.0005 (9)	0.0025 (9)	0.0011 (8)
C2	0.0250 (12)	0.0190 (11)	0.0202 (12)	−0.0016 (9)	0.0033 (10)	0.0023 (9)
C3	0.0221 (12)	0.0245 (12)	0.0191 (12)	0.0008 (9)	−0.0009 (10)	0.0019 (9)
C4	0.0211 (12)	0.0246 (11)	0.0219 (12)	−0.0038 (9)	0.0028 (10)	−0.0033 (9)
C5	0.0272 (13)	0.0216 (11)	0.0242 (12)	−0.0034 (9)	0.0025 (10)	0.0038 (9)
C6	0.0214 (12)	0.0264 (11)	0.0171 (11)	0.0024 (9)	0.0001 (9)	0.0008 (9)
C7	0.0186 (12)	0.0301 (13)	0.0271 (13)	0.0016 (10)	−0.0004 (10)	−0.0049 (10)
C8	0.0340 (15)	0.0240 (12)	0.0306 (14)	−0.0011 (11)	0.0021 (11)	−0.0023 (10)
C9	0.0308 (14)	0.0219 (11)	0.0301 (13)	−0.0002 (10)	0.0026 (11)	0.0050 (10)
C10	0.060 (2)	0.0535 (18)	0.0340 (16)	−0.0199 (15)	0.0169 (14)	−0.0058 (13)
C11	0.0211 (12)	0.0224 (11)	0.0212 (12)	−0.0003 (9)	0.0041 (10)	−0.0016 (9)
C12	0.0210 (12)	0.0209 (11)	0.0215 (12)	−0.0012 (9)	0.0035 (9)	0.0006 (9)
C13	0.0237 (12)	0.0184 (11)	0.0202 (12)	−0.0010 (9)	0.0039 (9)	−0.0001 (8)
C14	0.0197 (12)	0.0226 (11)	0.0186 (11)	0.0002 (9)	0.0024 (9)	−0.0017 (9)
C15	0.0209 (12)	0.0212 (12)	0.0215 (12)	−0.0023 (9)	0.0031 (10)	−0.0018 (9)
C16	0.0263 (13)	0.0184 (10)	0.0170 (11)	0.0005 (9)	0.0045 (9)	−0.0002 (9)
C17	0.0264 (13)	0.0200 (11)	0.0167 (12)	0.0031 (10)	0.0017 (9)	−0.0046 (9)
C18	0.0218 (12)	0.0234 (12)	0.0213 (12)	−0.0014 (9)	0.0031 (10)	−0.0021 (9)
C19	0.0252 (12)	0.0240 (12)	0.0175 (11)	−0.0032 (10)	0.0035 (9)	−0.0026 (9)
C20	0.0347 (15)	0.0288 (13)	0.0299 (14)	−0.0098 (11)	0.0019 (12)	0.0041 (10)
C21	0.0247 (13)	0.0295 (13)	0.0247 (13)	0.0034 (10)	−0.0044 (10)	−0.0021 (10)
C22	0.0335 (15)	0.0368 (15)	0.0245 (13)	0.0003 (12)	0.0012 (11)	−0.0058 (11)
C23	0.0235 (12)	0.0223 (11)	0.0211 (12)	0.0015 (9)	0.0015 (9)	0.0000 (9)
C24	0.0242 (13)	0.0217 (11)	0.0230 (12)	0.0013 (9)	0.0022 (10)	−0.0007 (9)
C25	0.0230 (12)	0.0250 (12)	0.0216 (12)	−0.0005 (10)	−0.0022 (10)	−0.0018 (9)
C26	0.0193 (12)	0.0263 (12)	0.0222 (12)	0.0010 (9)	0.0010 (9)	0.0036 (9)
C27	0.0250 (13)	0.0216 (11)	0.0219 (12)	0.0021 (9)	0.0029 (10)	−0.0022 (9)
C28	0.0248 (13)	0.0216 (11)	0.0178 (11)	−0.0028 (9)	0.0020 (9)	−0.0001 (8)
C29	0.0186 (12)	0.0276 (12)	0.0317 (14)	0.0008 (10)	−0.0030 (10)	0.0000 (10)
C30	0.063 (2)	0.0250 (13)	0.0297 (14)	0.0034 (13)	0.0042 (14)	0.0063 (11)
C31	0.0282 (14)	0.0279 (12)	0.0246 (13)	0.0033 (10)	0.0028 (11)	−0.0073 (10)
C32	0.0335 (16)	0.0358 (14)	0.0536 (19)	−0.0032 (12)	0.0074 (13)	−0.0035 (13)
C33	0.0236 (12)	0.0232 (12)	0.0235 (12)	0.0030 (10)	0.0022 (10)	0.0002 (9)
C34	0.0242 (13)	0.0269 (12)	0.0217 (12)	0.0035 (10)	0.0022 (10)	0.0005 (9)
C35	0.0232 (12)	0.0186 (11)	0.0203 (12)	0.0002 (9)	0.0034 (9)	0.0001 (9)
C36	0.0194 (12)	0.0213 (11)	0.0168 (11)	−0.0020 (9)	0.0030 (9)	0.0015 (8)
C37	0.0233 (12)	0.0211 (11)	0.0191 (12)	0.0012 (9)	0.0050 (9)	−0.0010 (9)
C38	0.0162 (11)	0.0229 (11)	0.0228 (12)	0.0035 (9)	0.0031 (9)	0.0020 (9)
C39	0.0174 (11)	0.0227 (11)	0.0174 (11)	−0.0033 (9)	0.0015 (9)	0.0022 (9)
C40	0.0239 (12)	0.0156 (10)	0.0189 (11)	−0.0021 (9)	0.0075 (9)	−0.0004 (8)
C41	0.0175 (11)	0.0193 (11)	0.0249 (12)	0.0018 (9)	0.0043 (9)	0.0027 (9)
C42	0.0374 (15)	0.0249 (12)	0.0285 (14)	0.0083 (11)	0.0074 (12)	−0.0050 (10)
C43	0.0190 (12)	0.0306 (13)	0.0242 (13)	−0.0009 (10)	−0.0011 (10)	−0.0010 (10)
C44	0.0316 (14)	0.0289 (13)	0.0284 (14)	0.0000 (11)	0.0012 (11)	0.0031 (10)

### Geometric parameters (Å, °)

**Table:**

O1—C4	1.367 (3)	C14—C19	1.389 (3)
O1—C7	1.432 (3)	C14—C15	1.406 (3)
O2—C7	1.385 (3)	C15—C16	1.380 (3)
O2—C8	1.433 (3)	C15—H15	0.9500
O3—C6	1.375 (3)	C16—C17	1.409 (3)
O3—C9	1.440 (3)	C17—C18	1.389 (3)
O4—C9	1.384 (3)	C18—C19	1.381 (3)
O4—C10	1.436 (3)	C18—H18	0.9500
O5—C2	1.353 (3)	C19—H19	0.9500
O5—H5	0.8400	C20—H20A	0.9800
O6—C11	1.256 (3)	C20—H20B	0.9800
O7—C16	1.368 (2)	C20—H20C	0.9800
O7—C20	1.431 (3)	C21—H21A	0.9900
O8—C17	1.370 (3)	C21—H21B	0.9900
O8—C21	1.428 (3)	C22—H22A	0.9800
O9—C21	1.391 (3)	C22—H22B	0.9800
O9—C22	1.437 (3)	C22—H22C	0.9800
O10—C26	1.366 (3)	C23—C24	1.430 (3)
O10—C29	1.423 (3)	C23—C28	1.433 (3)
O11—C29	1.385 (3)	C23—C33	1.468 (3)
O11—C30	1.430 (3)	C24—C25	1.386 (3)
O12—C28	1.373 (3)	C25—C26	1.376 (3)
O12—C31	1.436 (2)	C25—H25	0.9500
O13—C31	1.390 (3)	C26—C27	1.395 (3)
O13—C32	1.420 (3)	C27—C28	1.373 (3)
O14—C24	1.347 (3)	C27—H27	0.9500
O14—H14	0.8400	C29—H29A	0.9900
O15—C33	1.261 (3)	C29—H29B	0.9900
O16—C40	1.371 (2)	C30—H30A	0.9800
O16—C42	1.424 (3)	C30—H30B	0.9800
O17—C39	1.367 (2)	C30—H30C	0.9800
O17—C43	1.427 (3)	C31—H31A	0.9900
O18—C43	1.395 (3)	C31—H31B	0.9900
O18—C44	1.432 (3)	C32—H32A	0.9800
C1—C2	1.419 (3)	C32—H32B	0.9800
C1—C6	1.430 (3)	C32—H32C	0.9800
C1—C11	1.468 (3)	C33—C34	1.473 (3)
C2—C3	1.387 (3)	C34—C35	1.328 (3)
C3—C4	1.376 (3)	C34—H34	0.9500
C3—H3	0.9500	C35—C36	1.463 (3)
C4—C5	1.406 (3)	C35—H35	0.9500
C5—C6	1.367 (3)	C36—C37	1.383 (3)
C5—H5A	0.9500	C36—C41	1.410 (3)
C7—H7A	0.9900	C37—C38	1.393 (3)
C7—H7B	0.9900	C37—H37	0.9500
C8—H8A	0.9800	C38—C39	1.389 (3)
C8—H8B	0.9800	C38—H38	0.9500
C8—H8C	0.9800	C39—C40	1.414 (3)
C9—H9A	0.9900	C40—C41	1.383 (3)
C9—H9B	0.9900	C41—H41	0.9500
C10—H10A	0.9800	C42—H42A	0.9800
C10—H10B	0.9800	C42—H42B	0.9800
C10—H10C	0.9800	C42—H42C	0.9800
C11—C12	1.479 (3)	C43—H43A	0.9900
C12—C13	1.335 (3)	C43—H43B	0.9900
C12—H12	0.9500	C44—H44A	0.9800
C13—C14	1.462 (3)	C44—H44B	0.9800
C13—H13	0.9500	C44—H44C	0.9800
			
C4—O1—C7	118.07 (18)	O8—C21—H21B	108.9
C7—O2—C8	112.09 (17)	H21A—C21—H21B	107.7
C6—O3—C9	118.27 (18)	O9—C22—H22A	109.5
C9—O4—C10	111.77 (18)	O9—C22—H22B	109.5
C2—O5—H5	109.5	H22A—C22—H22B	109.5
C16—O7—C20	116.70 (18)	O9—C22—H22C	109.5
C17—O8—C21	117.35 (18)	H22A—C22—H22C	109.5
C21—O9—C22	113.39 (19)	H22B—C22—H22C	109.5
C26—O10—C29	117.62 (18)	C24—C23—C28	115.58 (19)
C29—O11—C30	113.18 (18)	C24—C23—C33	118.2 (2)
C28—O12—C31	119.19 (17)	C28—C23—C33	126.1 (2)
C31—O13—C32	112.67 (19)	O14—C24—C25	116.0 (2)
C24—O14—H14	109.5	O14—C24—C23	121.6 (2)
C40—O16—C42	116.60 (18)	C25—C24—C23	122.5 (2)
C39—O17—C43	117.11 (17)	C26—C25—C24	119.1 (2)
C43—O18—C44	113.86 (17)	C26—C25—H25	120.5
C2—C1—C6	115.69 (19)	C24—C25—H25	120.5
C2—C1—C11	119.03 (19)	O10—C26—C25	124.4 (2)
C6—C1—C11	125.28 (19)	O10—C26—C27	114.43 (19)
O5—C2—C3	116.5 (2)	C25—C26—C27	121.2 (2)
O5—C2—C1	120.8 (2)	C28—C27—C26	120.1 (2)
C3—C2—C1	122.7 (2)	C28—C27—H27	119.9
C4—C3—C2	118.8 (2)	C26—C27—H27	119.9
C4—C3—H3	120.6	C27—C28—O12	121.77 (19)
C2—C3—H3	120.6	C27—C28—C23	121.5 (2)
O1—C4—C3	125.0 (2)	O12—C28—C23	116.70 (19)
O1—C4—C5	113.91 (19)	O11—C29—O10	114.05 (19)
C3—C4—C5	121.1 (2)	O11—C29—H29A	108.7
C6—C5—C4	119.6 (2)	O10—C29—H29A	108.7
C6—C5—H5A	120.2	O11—C29—H29B	108.7
C4—C5—H5A	120.2	O10—C29—H29B	108.7
C5—C6—O3	122.4 (2)	H29A—C29—H29B	107.6
C5—C6—C1	121.8 (2)	O11—C30—H30A	109.5
O3—C6—C1	115.75 (19)	O11—C30—H30B	109.5
O2—C7—O1	112.93 (18)	H30A—C30—H30B	109.5
O2—C7—H7A	109.0	O11—C30—H30C	109.5
O1—C7—H7A	109.0	H30A—C30—H30C	109.5
O2—C7—H7B	109.0	H30B—C30—H30C	109.5
O1—C7—H7B	109.0	O13—C31—O12	112.08 (18)
H7A—C7—H7B	107.8	O13—C31—H31A	109.2
O2—C8—H8A	109.5	O12—C31—H31A	109.2
O2—C8—H8B	109.5	O13—C31—H31B	109.2
H8A—C8—H8B	109.5	O12—C31—H31B	109.2
O2—C8—H8C	109.5	H31A—C31—H31B	107.9
H8A—C8—H8C	109.5	O13—C32—H32A	109.5
H8B—C8—H8C	109.5	O13—C32—H32B	109.5
O4—C9—O3	112.51 (18)	H32A—C32—H32B	109.5
O4—C9—H9A	109.1	O13—C32—H32C	109.5
O3—C9—H9A	109.1	H32A—C32—H32C	109.5
O4—C9—H9B	109.1	H32B—C32—H32C	109.5
O3—C9—H9B	109.1	O15—C33—C23	119.3 (2)
H9A—C9—H9B	107.8	O15—C33—C34	117.2 (2)
O4—C10—H10A	109.5	C23—C33—C34	123.6 (2)
O4—C10—H10B	109.5	C35—C34—C33	121.1 (2)
H10A—C10—H10B	109.5	C35—C34—H34	119.4
O4—C10—H10C	109.5	C33—C34—H34	119.4
H10A—C10—H10C	109.5	C34—C35—C36	127.5 (2)
H10B—C10—H10C	109.5	C34—C35—H35	116.2
O6—C11—C1	119.6 (2)	C36—C35—H35	116.2
O6—C11—C12	118.4 (2)	C37—C36—C41	118.4 (2)
C1—C11—C12	121.93 (19)	C37—C36—C35	120.2 (2)
C13—C12—C11	120.7 (2)	C41—C36—C35	121.4 (2)
C13—C12—H12	119.6	C36—C37—C38	121.8 (2)
C11—C12—H12	119.6	C36—C37—H37	119.1
C12—C13—C14	128.0 (2)	C38—C37—H37	119.1
C12—C13—H13	116.0	C39—C38—C37	119.8 (2)
C14—C13—H13	116.0	C39—C38—H38	120.1
C19—C14—C15	118.2 (2)	C37—C38—H38	120.1
C19—C14—C13	119.2 (2)	O17—C39—C38	125.2 (2)
C15—C14—C13	122.6 (2)	O17—C39—C40	115.65 (19)
C16—C15—C14	121.0 (2)	C38—C39—C40	119.16 (19)
C16—C15—H15	119.5	O16—C40—C41	124.3 (2)
C14—C15—H15	119.5	O16—C40—C39	115.41 (19)
O7—C16—C15	124.5 (2)	C41—C40—C39	120.24 (19)
O7—C16—C17	115.6 (2)	C40—C41—C36	120.6 (2)
C15—C16—C17	119.9 (2)	C40—C41—H41	119.7
O8—C17—C18	125.0 (2)	C36—C41—H41	119.7
O8—C17—C16	115.94 (19)	O16—C42—H42A	109.5
C18—C17—C16	119.0 (2)	O16—C42—H42B	109.5
C19—C18—C17	120.4 (2)	H42A—C42—H42B	109.5
C19—C18—H18	119.8	O16—C42—H42C	109.5
C17—C18—H18	119.8	H42A—C42—H42C	109.5
C18—C19—C14	121.4 (2)	H42B—C42—H42C	109.5
C18—C19—H19	119.3	O18—C43—O17	113.16 (19)
C14—C19—H19	119.3	O18—C43—H43A	108.9
O7—C20—H20A	109.5	O17—C43—H43A	108.9
O7—C20—H20B	109.5	O18—C43—H43B	108.9
H20A—C20—H20B	109.5	O17—C43—H43B	108.9
O7—C20—H20C	109.5	H43A—C43—H43B	107.8
H20A—C20—H20C	109.5	O18—C44—H44A	109.5
H20B—C20—H20C	109.5	O18—C44—H44B	109.5
O9—C21—O8	113.23 (19)	H44A—C44—H44B	109.5
O9—C21—H21A	108.9	O18—C44—H44C	109.5
O8—C21—H21A	108.9	H44A—C44—H44C	109.5
O9—C21—H21B	108.9	H44B—C44—H44C	109.5
			
C6—C1—C2—O5	−176.5 (2)	C28—C23—C24—O14	179.8 (2)
C11—C1—C2—O5	4.1 (3)	C33—C23—C24—O14	3.1 (3)
C6—C1—C2—C3	4.6 (3)	C28—C23—C24—C25	0.3 (3)
C11—C1—C2—C3	−174.7 (2)	C33—C23—C24—C25	−176.4 (2)
O5—C2—C3—C4	−178.5 (2)	O14—C24—C25—C26	−178.03 (19)
C1—C2—C3—C4	0.4 (3)	C23—C24—C25—C26	1.5 (3)
C7—O1—C4—C3	−10.9 (3)	C29—O10—C26—C25	10.2 (3)
C7—O1—C4—C5	169.68 (18)	C29—O10—C26—C27	−170.93 (18)
C2—C3—C4—O1	176.7 (2)	C24—C25—C26—O10	177.4 (2)
C2—C3—C4—C5	−3.9 (3)	C24—C25—C26—C27	−1.4 (3)
O1—C4—C5—C6	−178.4 (2)	O10—C26—C27—C28	−179.44 (19)
C3—C4—C5—C6	2.1 (3)	C25—C26—C27—C28	−0.6 (3)
C4—C5—C6—O3	−175.10 (19)	C26—C27—C28—O12	−176.98 (19)
C4—C5—C6—C1	3.3 (3)	C26—C27—C28—C23	2.5 (3)
C9—O3—C6—C5	−3.8 (3)	C31—O12—C28—C27	4.3 (3)
C9—O3—C6—C1	177.69 (18)	C31—O12—C28—C23	−175.19 (18)
C2—C1—C6—C5	−6.5 (3)	C24—C23—C28—C27	−2.3 (3)
C11—C1—C6—C5	172.8 (2)	C33—C23—C28—C27	174.1 (2)
C2—C1—C6—O3	172.05 (18)	C24—C23—C28—O12	177.19 (19)
C11—C1—C6—O3	−8.7 (3)	C33—C23—C28—O12	−6.4 (3)
C8—O2—C7—O1	72.9 (2)	C30—O11—C29—O10	−67.2 (3)
C4—O1—C7—O2	83.6 (2)	C26—O10—C29—O11	−73.6 (2)
C10—O4—C9—O3	−65.3 (2)	C32—O13—C31—O12	−68.7 (2)
C6—O3—C9—O4	−67.5 (2)	C28—O12—C31—O13	−72.7 (2)
C2—C1—C11—O6	−14.3 (3)	C24—C23—C33—O15	4.5 (3)
C6—C1—C11—O6	166.5 (2)	C28—C23—C33—O15	−171.9 (2)
C2—C1—C11—C12	162.98 (19)	C24—C23—C33—C34	−176.9 (2)
C6—C1—C11—C12	−16.3 (3)	C28—C23—C33—C34	6.8 (3)
O6—C11—C12—C13	−13.5 (3)	O15—C33—C34—C35	6.1 (3)
C1—C11—C12—C13	169.2 (2)	C23—C33—C34—C35	−172.6 (2)
C11—C12—C13—C14	−178.7 (2)	C33—C34—C35—C36	178.6 (2)
C12—C13—C14—C19	−178.5 (2)	C34—C35—C36—C37	172.2 (2)
C12—C13—C14—C15	1.0 (4)	C34—C35—C36—C41	−7.6 (3)
C19—C14—C15—C16	1.6 (3)	C41—C36—C37—C38	1.5 (3)
C13—C14—C15—C16	−177.9 (2)	C35—C36—C37—C38	−178.38 (19)
C20—O7—C16—C15	−2.5 (3)	C36—C37—C38—C39	0.3 (3)
C20—O7—C16—C17	176.30 (19)	C43—O17—C39—C38	5.8 (3)
C14—C15—C16—O7	178.64 (19)	C43—O17—C39—C40	−174.90 (18)
C14—C15—C16—C17	−0.1 (3)	C37—C38—C39—O17	177.26 (19)
C21—O8—C17—C18	−4.0 (3)	C37—C38—C39—C40	−2.1 (3)
C21—O8—C17—C16	178.21 (19)	C42—O16—C40—C41	3.0 (3)
O7—C16—C17—O8	−3.0 (3)	C42—O16—C40—C39	−177.43 (19)
C15—C16—C17—O8	175.86 (18)	O17—C39—C40—O16	3.1 (3)
O7—C16—C17—C18	179.07 (18)	C38—C39—C40—O16	−177.50 (18)
C15—C16—C17—C18	−2.0 (3)	O17—C39—C40—C41	−177.32 (18)
O8—C17—C18—C19	−174.9 (2)	C38—C39—C40—C41	2.1 (3)
C16—C17—C18—C19	2.8 (3)	O16—C40—C41—C36	179.23 (19)
C17—C18—C19—C14	−1.3 (3)	C39—C40—C41—C36	−0.3 (3)
C15—C14—C19—C18	−0.8 (3)	C37—C36—C41—C40	−1.5 (3)
C13—C14—C19—C18	178.70 (19)	C35—C36—C41—C40	178.39 (19)
C22—O9—C21—O8	68.4 (2)	C44—O18—C43—O17	−66.9 (2)
C17—O8—C21—O9	66.4 (2)	C39—O17—C43—O18	−66.7 (2)

### Hydrogen-bond geometry (Å, °)

**Table:**

*D*—H···*A*	*D*—H	H···*A*	*D*···*A*	*D*—H···*A*
O5—H5···O6	0.84	1.75	2.506 (2)	148.
O14—H14···O15	0.84	1.72	2.475 (2)	148.
C8—H8C···O8^i^	0.98	2.57	3.312 (3)	132.
C9—H9A···O5^ii^	0.99	2.52	3.444 (3)	155.

Symmetry codes: (i) *x*−1, *y*, *z*−1; (ii) −*x*+1, *y*−1/2, −*z*+1.
